# Clinical Pathways as Instruments for Risk and Cost Management in Hospitals - A Discussion Paper

**DOI:** 10.5539/gjhs.v4n2p50

**Published:** 2012-03-01

**Authors:** Tobias Romeyke, Harald Stummer

**Affiliations:** Dept. of Public Health, Information Systems and Health Technology Assessment UMIT - University for Health Sciences, Medical Informatics and Technology Opernring 5, A-1010 Vienna, Austria

**Keywords:** Clinical pathway, Quality management, Multimodal pain therapy, Risk management, Cost efficiency, Total cost

## Abstract

**Introduction:**

The distinctive characteristics of the German health system are medical progress and financial pressure—and this is especially true of the hospitals. These challenges must be met by strategic management instruments for quality assurance, and by reducing costs.

**Purpose:**

This article presents the instrument “clinical pathway” (also known as “clinical treatment pathway”) and describes the possibilities it offers, both for quality assurance and risk management, and for cost reduction. The clinical pathway presented here will be that for “multimodal pain therapy”, as used in the context of acute inpatient care in Germany.

**Methods:**

A general presentation of the risks in hospital is followed by consideration of the risks associated with core processes. A comprehensive total cost analysis is performed for those patients who meet the pathway entry criteria and who fulfil the requirements for the structure of care provided within multimodal pain therapy.

**Discussion and Conclusion:**

Multimodal pain therapy places high demands on the structural, procedural and outcome quality of the medical, nursing and therapeutic services provided, and these demands are reflected in high costs for the provision of this care. The treatment process involves many different professional groups. These complex interfaces can potentially generate risks, which can lead to the possibility of legal liability. A clinical pathway must structure the core process and then combine elements of quality assurance in order to optimise patient care and minimise risk. The examination of costs reveals significant potential savings (patients with clinical pathway: EUR 3086±212; patients without clinical pathway: EUR 3774±460; Mann-Whitney U test; p<0.001). For the managers of a hospital, the clinical pathway represents a strategic management instrument that can serve for continual cost control and cost reduction, and can contribute in the form of quality assurance towards a transparent provision of services.

## 1. Introduction

### 1.1 Clinical Pathways

Health care world-wide presents a challenge regarding its organisation and the efficient allocation of resources. This challenge is increasing as a result of continual progress in the field of medicine and sustained demographic development. The greatest costs in the health system are generated by hospital treatment. The costs for the almost 2,100 hospitals in Germany increased by 6.1% during the year 2009 to reach a total of 77.1 billion euros (*Statistisches Bundesamt* “Federal Statistics Office”, 2011).

More and more hospitals in Germany are striving to restructure their procedures for providing services in order to generate potential for reducing costs. They are aiming simultaneously to optimise the quality of their processes and outcomes.

To this end, clinical pathways will become increasingly important in the context of case tariff fees as a part of the International Statistical Classification of Diseases and Related Health Problems (ICD) for inpatient hospital services. These pathways will contribute towards shortening the period of hospitalisation (Hommel *et al.*, 2008; Ishiguro *et al.*, 2008), reducing costs (Verdú *et al.*, 2009; Barbieri *et al.*, 2009; Rook 1998; Velasco *et al.*, 1996) and increasing the quality of the services provided (Schwarzbach *et al.*, 2010; Andriessen *et al.*, 2009; Feuth & Claes, 2008). A generally valid definition of “clinical pathway” exists neither in the USA, nor in Australia, nor in Europe. Hindle (1997) defines the clinical pathway as a document describing the services provided in the multidisciplinary treatment of a particular type of patient, which enables comments to be made about deviations from the norm for the purpose of evaluation and improvement.

The development of a clinical path must relate to indications or procedures and, in order to achieve greater efficiency and quality benefits, must cover the patients and cases focused on by the hospital and therefore occurring most frequently. Patients, doctors, nurses and therapeutic personnel must all be involved in the development of a clinical pathway in order to integrate indication-related, evidence-based and best-practice procedures as requirements in the clinical pathway. The present work examines in greater detail the contribution of a clinical pathway with regard to aspects of risk management, quality assurance and cost efficiency for multimodal pain therapy.

### 1.2 Multimodal Pain Therapy in Germany (OPS 8-918)

Physical pain is not the only symptom suffered by patients subject to chronic pain; at the psychological level, chronic pain is accompanied by a wide variety of cognitive and emotional factors. Patients suffering from chronic pain frequently suffer from accompanying diseases which can be manifested, for example, in the form of states of anxiety and panic, despair, depression and lability (Banks & Kerns 1996; McWilliams *et al.*, 2003). Most chronic pain patients withdraw from their social environment, show less and less interest in social contacts and activities, and become involved in interpersonal conflicts within their family, or among their friends, or colleagues at work (Cowan *et al.*, 1998).

The *Deutsche Gesellschaft zum Studium des Schmerzes (DGSS)* (“German Society for the Study of Pain”) defines multimodal pain therapy as the “interdisciplinary treatment of patients with chronic pain syndrome in such a way that the different therapies, given simultaneously with mutual coordination of their content, chronology and procedure, are integrated with the different somatic, physical, psychological, occupational and psychotherapeutic methods according to a predefined therapy plan with an identical therapeutic target agreed among the therapists”.

Multimodal pain therapy is listed as Procedure 8-918 in the *Operationen- und Prozedurenschlüssel (OPS)* (“Operations and Procedures Code”) in the versions published by the *Deutschen Institut für Medizinische Dokumentation* (“German Institute for Medical Documentation”) at the behest of the *Bundesministerium für Gesundheit* (“Federal Ministry of Health”) pursuant to §§295 and 301 *SGB* V (“Social Code”). The acceptance and further development of inpatient multimodal pain therapy within the German case tariff fee system (*G-DRG*) result from the efforts of a number of professional associations, which include the *Deutsche Gesellschaft zum Studium des Schmerzes (DGSS)* (“German Society for the Study of Pain”, the *Berufsverband Deutscher Anästhesisten (BDA)* (“Professional Association of German Anaesthetists”, the *Gesellschaft für Anästhesiologie und Intensivmedizin (DGAI)* (“Association for Anaesthesiology and Intensive Medicine”), and the *Deutsche Interdisziplinäre Vereinigung für Schmerztherapie (DIVS)* (“German Interdisciplinary Association for Pain Therapy”). The multimodal pain therapy is used in specialized clinics in Germany.

Multimodal pain therapy in accordance with *OPS* 8-918 places high demands on the structural, procedural and outcome quality of the provision of inpatient services. The *OPS* stipulates an interdisciplinary team whose members have specialised in different fields. The therapy manager must be a specialist with an additional qualification in “special pain therapy”. The term special pain therapy requires special knowledge in the areas of conservative medical (eg, internal medicine, neurology, etc.), operative medicine (surgery, neurosurgery, orthopedics, etc.) and conservative and interventional medicine (anesthesiology, radiation, etc.). Particulary basic knowledge about the pathogenesis, diagnosis and therapy, bio-psycho-social history of pain, mental disorders with pain and psychosomatic interactions in chronic pain states further expertise in neuropathic pain, pain with vascular disease, pain in visceral disease, cancer pain, pain in children and adolescents, muscle pain, back pain, joint disorders. The structural requirements for performing multimodal pain therapy also correspond with the requirements of evidence-based provision of services. According to this, indication-related behavioural therapy (Chou & Huffman 2007; Gatchel & Rollings, 2008), occupational therapy with movement-therapy approaches (Hayden *et al.*, 2005) and ergotherapeutic methods (Williams *et al.*, 2007) must be integrated in the therapy plan. Ergotherapeutic methods include “joint protection measures”, “learning of substitute functions”, “improving the mobility and locomotion”, “hand therapy”, “training of life skills”.

Depending on the clinical picture, physiotherapeutic measures (Gross *et al.*, 2004), art (Sexton-Radek, 1999) and music therapy (Good *et al.*, 2002; Tan *et al.*, 2010), therapy for workplace training, sensomotoric training, and medical training therapy (Valim *et al.*, 2003) must be prescribed by the medical staff. Therapies for worplace training include for example correct posture, stretching and relaxation exercises at the PC workstation.

The prescribed treatment methods are used depending on the clinical picture. The methods of conventional medicine are transformed into an integrated and holistic approach of therapy.

## 2. Methods

### 2.1 Risks in the Provision of Inpatient Services

As a result of legal requirements and the fact that human life is at the focus of the services provided, the risks prevalent in a hospital are particularly diverse. Starting from the general risks in the hospital, attention must be directed towards the procedure-related risks, and approaches to solutions must be worked out for multimodal pain therapy.

### 2.2 Pathway Entry Criteria

**Figure 1:**
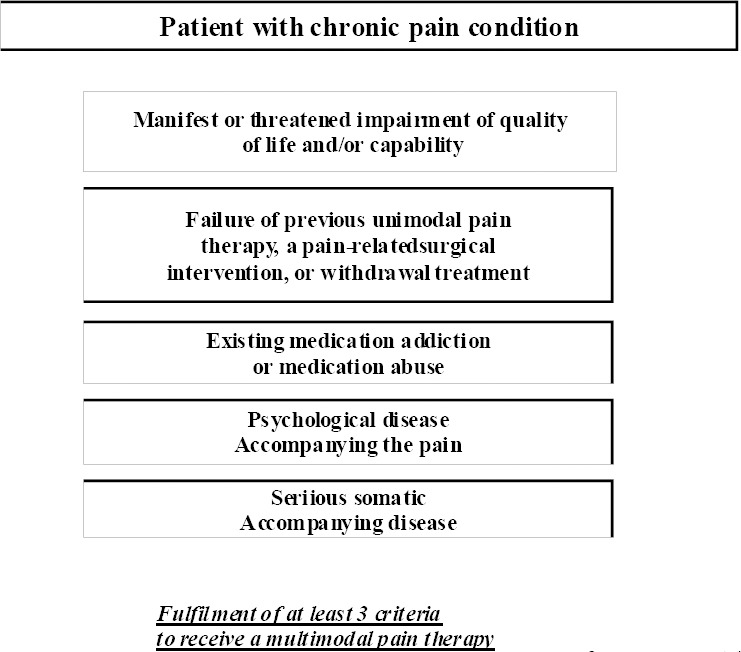
Pathway entry criteria for multimodal pain therapy (OPS 8-918)

Examination of the pathway entry criteria (Fig. 1) is an integral part of structured admission management, based on which the therapeutic objectives are defined and the pathway sequence is oriented; this, in turn, demands complex interface management.

### 2.3 Structural Requirements and Provision of Services

The code for multimodal pain therapy stipulates a minimum of seven days interdisciplinary treatment (by legislation) of patients with chronic pain conditions (including tumour pain) with involvement of at least two specialist fields (of which one must be a psychiatric, psychosomatic or psychological discipline). In addition, at least three of the following active therapeutic methods must be used simultaneously: psychotherapy, physiotherapy, relaxation techniques, ergotherapy, medical training therapy, sensomotoric training, workplace training, artistic therapy (art or music therapy) or other kinds of occupational therapy. The therapy sessions last an average of 30 minutes. The code also includes an evaluation of the progression of the treatment by means of a standardised therapeutic assessment, a daily doctor’s visit or team discussion, and a weekly interdisciplinary team meeting. In group therapy, the size of the group is limited to a maximum of 8 people. Use of this code requires that the responsible doctor has the additional qualification “special pain therapy” (*OPS* 8-918; The operations and procedure code (*OPS*) is published by DIMDI [“German Institute for Medical Documentation”] on behalf of the Federal Ministry of Health).

### 2.4 Costs Analysis

Based on the path entry criteria (Section 2.2.) and the structural requirements for providing services (Section 2.3.), an analysis was performed of the total costs incurred by 65 subjects who received inpatient treatment in accordance with the requirements of multimodal pain therapy. The costs data were acquired by means of cost type accounting, cost centre accounting by means of cost-centre related cost distribution, and differential in-house performance accounting which localises the places at which costs are incurred. As the final stage of the cost accounting, cost unit accounting identifies the reasons for the costs incurred and allocates the final cost centres to the individual beneficiaries of services.

## 3. Discussion and Conclusion

### 3.1 Path-Indicated Quality Assurance and Risk Minimisation

Hospitals are characterised by complex interfaces.

The risks to which a hospital is exposed are extremely complex, at the level of both the control processes and the support processes, and care is essential when examining them (Fig. 3). Control processes in the hospital include strategic management and controlling, and the requirements for internal and external quality assurance. Support processes include financial controlling, materials management and pharmaceutical services.

**Figure 2:**
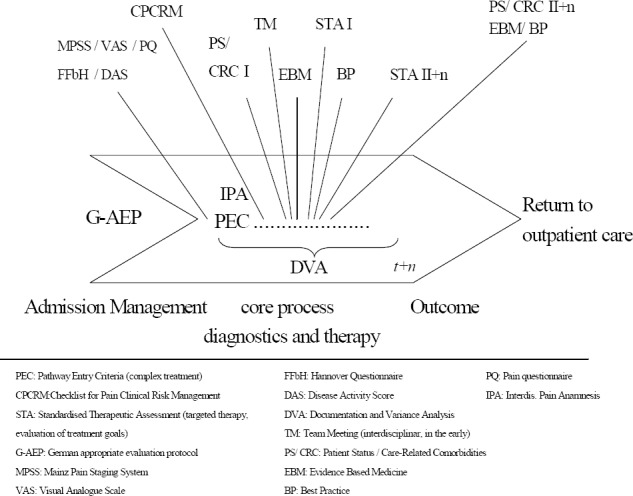
Clinical Pathway: overview pain therapy

**Figure 3:**
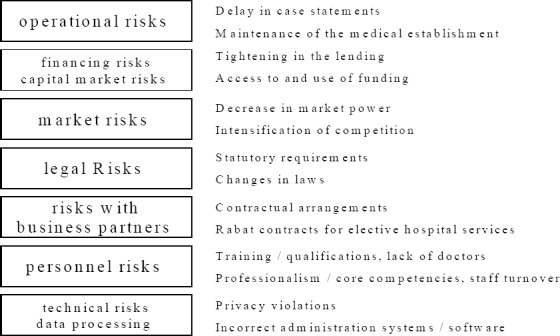
Risks in the hospital at the organizational level

German legislation ensures greater safeguards for stake- and shareholders in that it defines appropriate standards for issuing early warnings of risks that could lead to the demise of the enterprise. This includes the *Gesetz zur Kontrolle und Transparenz im Unternehmensbereich (KonTraG)* (“Corporate Sector Supervision and Transparency Act”). The core of the *KonTraG* is a regulation that obligates the company management to implement a risk early-warning system and which extends the liability of the board of directors, supervisory board and auditors. Against a background of increasing liability claims for errors in treatment, risk management also occupies an important position at the core process level (diagnosis and therapy).

According to Führing and Gausmann (2004), the aims of clinical risk management are the continual improvement of treatment quality and patient safety, and defence of the hospital against unjustified claims against the institution made by patients (Führing & Gausmann, 2004).

Studies have shown that the number of cases in which patients suffer harm while receiving treatment is considerable (Thomas *et al.*, 2000; Weingart *et al.*, 2000; Baker *et al.*, 2004). An analysis of 1014 treatment cases selected at random in two English hospitals revealed that 10.8% of the patients suffered unintentional impairment to their health; 46% of the errors could potentially have been avoided (Vincent & Neale, 2001). Organisational deficiencies are especially prevalent as the (co-)origin of accusations of error (Hansis & Hansis, 1999). Inadequate documentation and education, insufficient communication between members of the individual professions and failure to comply with departmental operating procedures can also contribute to the poor progression of treatment, and to accusations of error.

Risk management and quality management are inextricably connected and, as an integrated system (Roeder *et al.*, 2007), pursue the objective of minimising the risks for patients, staff and the organisation, and maximising the quality of the care provided (Bernsmann *et al.*, 2002).

**Figure 4:**
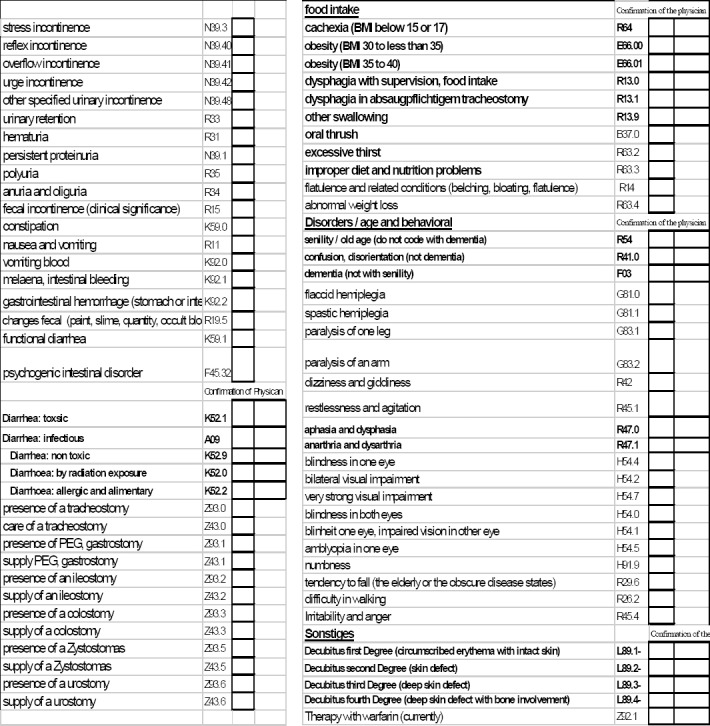
Performance record sheet of care: care-related comorbidities

Interdisciplinary diagnosis and treatment of pain involves many different professions. Complex interfaces can generate potential risks, which may lead to possible legal liability. In addition to the significance of possible liability, the occurrence of liability cases can result in patients’ dissatisfaction becoming publicly known which, in turn, can result in fewer patients and lower returns. Risk potentials must be identified in order to prevent liability cases. In the treatment progression of multimodal pain therapy, a clinical pathway stipulates standardisation of the multidisciplinary processes and services, enabling quality fluctuations to be reduced and treatment risks to be minimised (Schlüter *et al.*, 2006). Integration in the clinical pathway of all staff involved in the treatment process, and coordination of the clinical progression offers the possibility of overcoming interface problems. Clinical pathway *OPS* 8-918 stipulates an interdisciplinary pain anamnesis by the doctors, nurses and therapists (Fig.2), which includes not only a complete description of the patient’s past pain, but also of his or her present symptoms. In addition, possible care-relevant secondary diagnoses are made by the nursing staff (Fig.4) and evaluated and dealt with jointly with the other staff involved in the treatment process. This also complies with the principle of a holistic therapy of the patient’s pain.

The specialist medical evaluation of the pain screening and the other examinations define the type and sequence of the subsequent diagnoses and the medication and therapeutic regimen. Both the generation of findings for purposes of quality assurance and risk minimisation, and the monitoring of the therapeutic progression are subject to different score parameters specified according to indication, and these accompany the treatment processes. For example, the clinical pathway for multimodal pain therapy for diseases and disturbances of the musculoskeletal system and connective tissue requires the measurement, several times a day, of the pain intensity by means of the visual analogue scale (VAS) (Winlkelmann & Schreiber, 1997), whereas the functional capacity of patients with rheumatic diseases is recorded using the “Hannover Questionnaire” (FFbH) (Kohlmann & Raspe, 1996). The visual analog scale is a semiquantitive procedure for the subjective measurement of pain intensity. 12 statements by the FFbH allows to measure the functional capacity at activities of daily living (mobility, personal hygiene, dressing and undressing) which may be affected by problems in the spine. This instrument, according to Kohlmann and Raspe (1996) already light up moderate functional limitations found in back pain patients. On admission, all aspects of pain occurrence, pain sensitivity, social anamnesis, pain intensity, pain character and pain localisation are evaluated using the pain questionnaire of the *DGSS* (“German Society for the Study of Pain”). For inflammatory rheumatic diseases, the disease activity score (DAS) is used (Cruyssen *et al.*, 2005; Prevoo *et al.*, 1995) (Fig.2). This disease activity score indicates how high the disease activity is and was developed in Europe as an alternative to the ACR (American College of Rheumatology) criteria. Every diagnostic procedure functions as a risk management instrument (drug administration, determination of active and passive therapeutic measures, etc.) and serves at the same time to assure the quality of the process and the outcome.

In addition, all risks that arise in association with drugs administered for specific kinds of treatment and with methods of pain therapy must be recorded and discussed during a patient briefing.

The treatment process is accompanied by complete medical and therapeutic documentation and a variance analysis. The resulting transparency for the different providers of services forms the basis for minimisation of treatment risks and poor outcomes, maximisation of internal quality, and compliance with the requirements of quality assurance (Romeyke, 2009).

**Figure 5:**
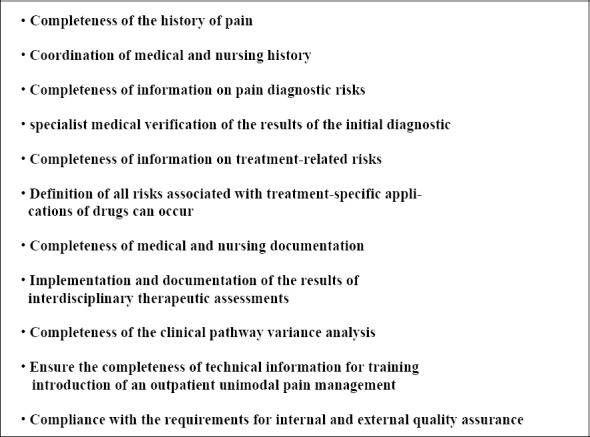
Checklist for Pain Clinical Risk Management

Figure 5 summarises as a checklist in eleven items the important elements of risk minimisation and quality assurance in pain therapy.

### 3.2 Generation of Efficiency Potentials

In addition to aspects of risk and quality management, active process management should also take cost aspects into account.

All 65 subjects fulfilled the pathway entry criteria described under 2.2., enabling Procedure 8-918 to be coded correctly in accordance with the requirements of the *OPS*.

The mean total costs for the 65 patients amounted to EUR 3467.08 (Fig. 6a and 6b). Patients for whom a clinical pathway (CP) was used incurred significantly lower costs than patients without CP (patients with CP: EUR 3086±212; patients without CP: EUR 3774±460; Mann-Whitney U test; p<0.001). This difference can be attributed to the clinical pathway requiring pre-defined, structured admission management with integrated pain anamnesis, the standardised use of evidence-based and best-practice measures, and the avoidance of inappropriate prescriptions. A clinical pathway must be designed so as to guarantee efficient allocation of resources in inpatient care.

**Figure 6a:**
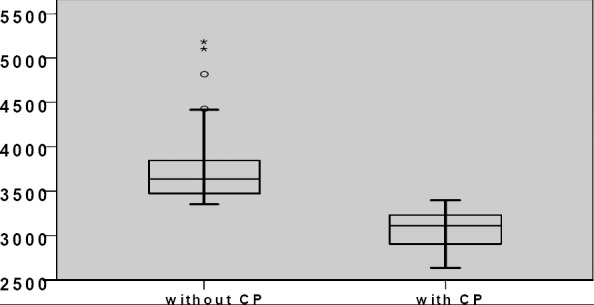
Total cost for patients with vs. without CP in (A€

**Figure 6b:**
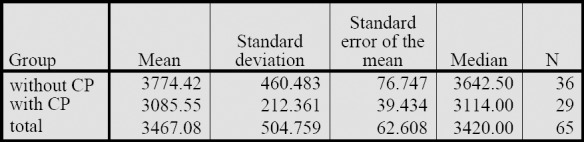
Total cost for patients with vs. without CP in €

### 3.3 Conclusion

Hospitals in future will have to cope with the fact that under the terms of a case tariff fee system, more cases will have to be treated with the same, or even a reduced, staffing level. It therefore appears necessary to identify and remove existing deficiencies in how procedures are organised. In the context of operative risk management, this must include the analysis, evaluation and monitoring of risks associated with diagnoses and procedures, with the aim of ensuring risk-free provision of services (Erben & Romeike, 2003). For the providers of services at the core process level (medical specialists, nursing staff, therapists and non ward-based nursing staff), clinical pathways will simplify the treatment process, and prevent—or at least minimise—risks by means of structured and comprehensive diagnosis and treatment procedures. Clinical pathways serve for quality assurance and make a contribution to ensuring net income from case tariff fees. By means of the indication- and procedure-related progression planning, they serve doctors, nurses and therapists as an instrument for the familiarisation and evaluation of the treatment process.

For the business management of a hospital, the clinical pathway presents a strategic management instrument that also serves as an instrument for continual cost controlling, and can contribute to transparency in the provision of services. Knowledge relevant to quality and to supply planning can be acquired and the range of services can be standardised without neglecting the individual requirements of the patients.
